# Oriented attachment explains cobalt ferrite nanoparticle growth in bioinspired syntheses

**DOI:** 10.3762/bjnano.5.23

**Published:** 2014-02-28

**Authors:** Annalena Wolff, Walid Hetaba, Marco Wißbrock, Stefan Löffler, Nadine Mill, Katrin Eckstädt, Axel Dreyer, Inga Ennen, Norbert Sewald, Peter Schattschneider, Andreas Hütten

**Affiliations:** 1 Department of Physics, Bielefeld University, 33615 Bielefeld, Germany; 2 (USTEM) University Service Centre for Transmission Electron Microscopy, Vienna University of Technology, 1040 Vienna, Austria; 3 Department of Chemistry, Bielefeld University, 33615 Bielefeld, Germany; 4 Institute Advanced Cemarics, Hamburg University of Technology, 21073 Hamburg, Germany; 5 Institute of Solid State Physics, Vienna University of Technology, 1040 Vienna, Austria

**Keywords:** bioinspired synthesis, cobalt ferrite nanoparticles, nanoparticle growth, oriented attachment, polypeptide

## Abstract

Oriented attachment has created a great debate about the description of crystal growth throughout the last decade. This aggregation-based model has successfully described biomineralization processes as well as forms of inorganic crystal growth, which could not be explained by classical crystal growth theory. Understanding the nanoparticle growth is essential since physical properties, such as the magnetic behavior, are highly dependent on the microstructure, morphology and composition of the inorganic crystals. In this work, the underlying nanoparticle growth of cobalt ferrite nanoparticles in a bioinspired synthesis was studied. Bioinspired syntheses have sparked great interest in recent years due to their ability to influence and alter inorganic crystal growth and therefore tailor properties of nanoparticles. In this synthesis, a short synthetic version of the protein MMS6, involved in nanoparticle formation within magnetotactic bacteria, was used to alter the growth of cobalt ferrite. We demonstrate that the bioinspired nanoparticle growth can be described by the oriented attachment model. The intermediate stages proposed in the theoretical model, including primary-building-block-like substructures as well as mesocrystal-like structures, were observed in HRTEM measurements. These structures display regions of substantial orientation and possess the same shape and size as the resulting discs. An increase in orientation with time was observed in electron diffraction measurements. The change of particle diameter with time agrees with the recently proposed kinetic model for oriented attachment.

## Introduction

Nanoparticles with a well-controlled microstructure, morphology and composition are essential for biomedical and magnetic recording applications [1–2]. These characteristics, which determine the physical properties such as the magnetic behavior, are highly sensitive to the crystal growth process. Understanding the growth mechanism is therefore neccessary if the physical properties of nanoparticles are to be tailored. Inorganic crystal formation and growth have been described via classical crystal growth theory for the past 100 years [3]. In this theory, crystal growth occurs via atom by atom (or monomer by monomer) addition to the crystal, with monomers as the smallest aggregates from which nuclei form. Coarsening, in which smaller crystals dissolve in favor of bigger ones, often occurs at later stages of the crystal growth. Throughout the last decades, several studies of nanoparticle growth and biomineralization processes showed a different underlying growth mechanism which cannot be explained by the classical growth theory [3–7].

**Figure 1:**
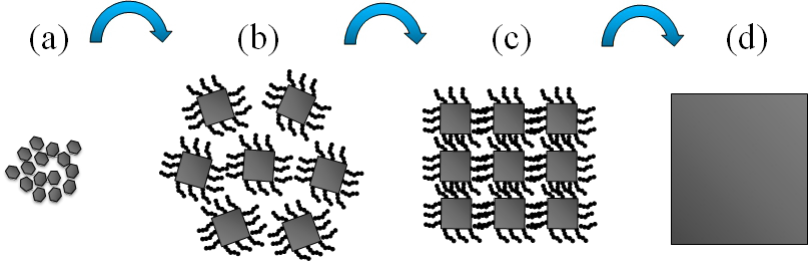
Schematic of the oriented attachment process that occurs in the presence of organic additives. (a) Nuclei grow into primary building blocks, which are displayed in (b). These primary building blocks reorganize and form a mesocrystal (c). The primary building blocks fuse at specific crystallographic faces and form a secondary nanoparticle, which is displayed in (d).

In 1998, Penn and Banfield introduced a new model called “oriented attachment” [8]. In this non-classical growth theory, crystal growth is dominated by kinetics [9]. Figure 1 gives an overview of the oriented attachment process. Individual crystals, called primary building blocks, self-assemble into metastable, intermediate phases such as mesocrystals [9–11], for which several formation mechanisms have been proposed. These mechanisms are summarized, e.g., in Cölfen’s work [7,11]. Mesocrystals display similar sizes and shapes as their aggregates which can either be iso-oriented crystals or single crystals. These aggregates are formed in the self-assembly of the building blocks (and their fusion in case of the single crystal) [11–12] and are called secondary nanoparticles [12]. The metastable, intermediate phase possesses a large amount of energetically unfavourable inner surfaces. To reduce the surface energy, adjacent primary building blocks can align along a common crystallographic axis and coalesce [12–13]. Phase transformations are often observed prior to aggregation [12]. The secondary nanoparticle, formed by this aggregation, often displays unusual morphologies [12] as well as regions of substantial orientation [14]. A more detailed description of this non-classical growth theory is given elsewhere [7–9,11–12,14–15]. Previous work has focussed on high resolution transmission electron microscopy measurements, in which defects and lattice fringes were studied to show the underlying aggregation-based growth process [14,16–19]. However, a complete growth process via oriented attachment, with all intermediate, metastable phases, has not yet been reported to our knowledge.

In this work, the underlying non-classical growth process of the biosynthesized cobalt ferrite nanoparticles, discussed in our previous work, was investigated [20]. Biomimetic approaches aim to specifically tailor particle properties under mild conditions, which cannot be achieved with conventional chemical bottom-up syntheses under similar conditions [21]. In these biomimetic syntheses, peptides are used which influence the inorganic crystal growth by different mechanisms, such as catalysis or surface adsorption. It was previously shown that c25-mms6, a short synthetic version of the protein MMS6 found in magnetotactic bacteria, can be used to obtain stoichiometric and shape specific cobalt ferrite nanoparticles [20,22]. The growth process, however, has remained elusive. Here, the composition, morphology and microstructure of the bioinspired synthesized nanoparticles were studied at different stages of the growth process using transmission electron microscopy (TEM), high resolution transmission electron microscopy (HRTEM), electron energy loss spectroscopy (EELS) and electron diffraction measurements.

## Results

**Figure 2:**
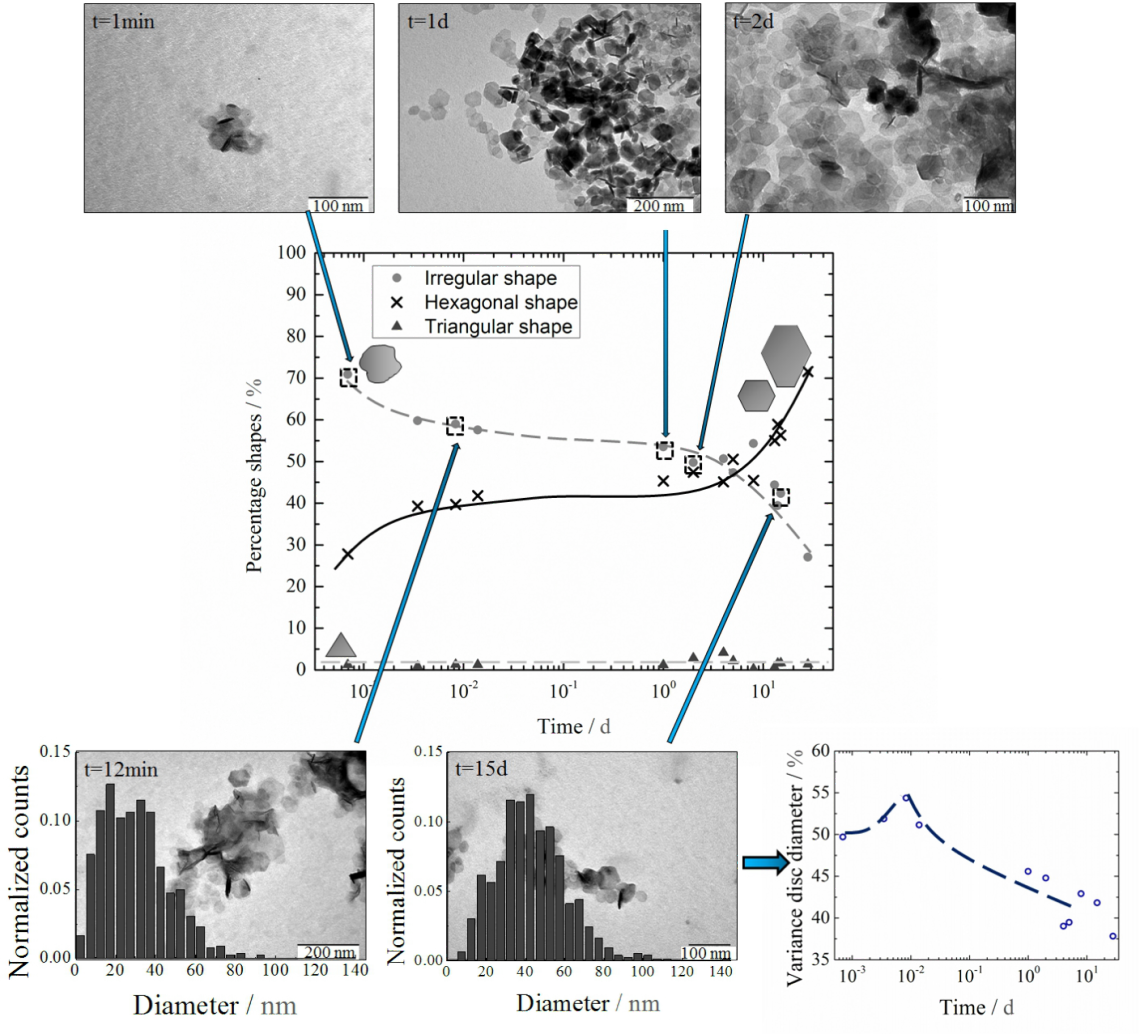
(a)–(c) Change in the electron diffraction pattern (shown with inverted intensity for better visibility) of diamond shaped particles with time. The dominating reflexes and rings are marked and indexed. (a) *t* = 5 min, (b) *t* = 1 d, (c) *t* = 2 d. (d) Bright field and dark field images of an incomplete diamond-shaped particle as well as the corresponding electron diffraction pattern of the disc. The highlighted areas in the dark field image correspond to the marked reflex of the electron diffraction pattern. (e) Image of an incomplete irregularly-shaped particle. The inset shows an enlarged area within the disc revealing that it is composed of smaller subunits. (f) HRTEM image of a final, stoichiometric monocrystalline disc, obtained after 28 days. The inset shows that the final nanoparticles are not porous.

In this bioinspired synthesis, stoichiometric Co_2_FeO_4_ discs of hexagonal, diamond-like, triangular or irregular shapes, and small stoichiometric CoFe_2_O_4_ spheres were obtained after 28 days. In addition incomplete non-stoichiometric discs were observed throughout the growth process. The different disc shapes observed in the TEM measurements are sketched in the graph located in the middle section of Figure 2. The stoichiometric iron-rich spheres (CoFe_2_O_4_) are not considered here, since they only form as a side product after 12 minutes due to the choice of the starting composition. The starting ratio of cobalt to iron was chosen to 1:2 to allow for a comparison to our previous work [20]. Since the discs are of a cobalt rich composition, the remaining iron precursor in the solution forms the side product (CoFe_2_O_4_).

**Figure 3:**
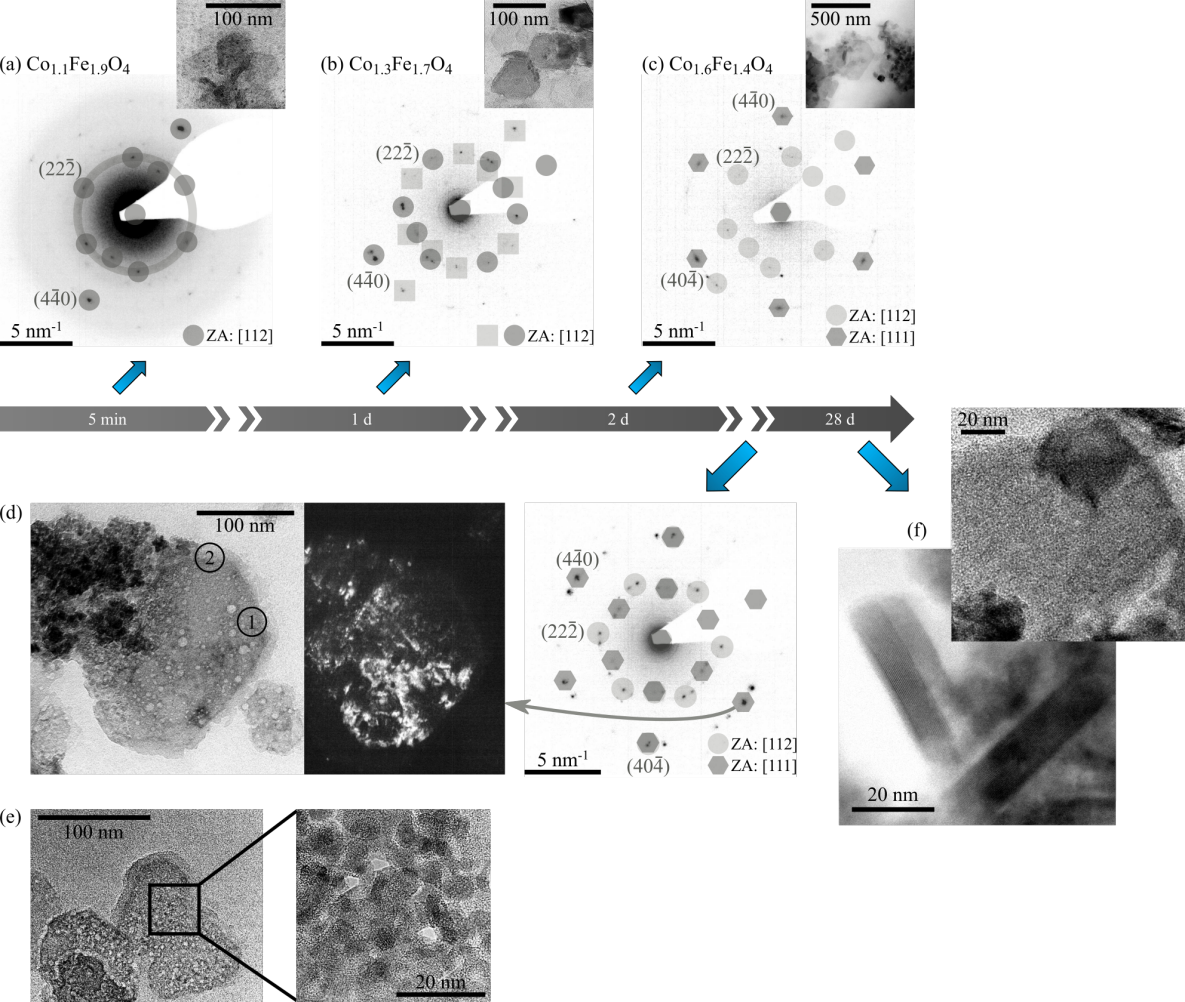
Top: Change in the electron diffraction pattern of diamond shaped particles with time. The inverted electron diffraction pattern is given here. (a) *t* = 5 min, (b) *t* = 1 d, (c) *t* = 2 d. Bottom: HRTEM images of incomplete particles. (d) Dark field image of an incomplete diamond-shaped particle as well as the corresponding electron diffraction pattern of the disc. The highlighted areas in the dark field image correspond to the specific reflex of the electron diffraction pattern. The inset of (e) shows an enlarged area within the disc. This disc is composed of smaller subunits.(f) HRTEM image of a final, stoichiometric monocrystalline disc, obtained after 28 days. The inset shows that the final nanoparticles are not porous. Only the dominating reflexes are indexed for reasons of clarity.

The particles at different stages of the growth process are displayed in Figure 2. EELS and TEM measurements of the nanoparticles at early stages of the growth process (from 5 minutes to 1 day) show that the initially formed discs are predominantly irregularly shaped (75%) and of iron-rich non-stoichiometric phases (see Figure 2 and Table 1). These discs display the (112) top/bottom crystal face which was determined by indexing the zone axis (ZA) of the electron diffraction patterns in Figure 3. The initial average particle diameter was determined to *D*_av_ = 29 nm, which remains unchanged during the first 20 minutes of particle growth. The size distribution at the early stages of particle growth (12 minutes, see Figure 2 (bottom)) shows a superposition of two maxima: one at *D*_small_ = 5–20 nm and a second one at *D*_large_ = 35 nm.

Incomplete discs, as displayed in Figure 3d,e can be found in addition to the discs throughout the entire growth process. HRTEM measurements of an incomplete, irregularly-shaped particle (Figure 3e) reveal that it is composed of several smaller irregularly-shaped subunits with diameters in the range of *D* = 5–15 nm. To study the orientation of these subunits a dark field measurement was performed. The marked reflex in the electron diffraction pattern (Figure 3d) was selected using the objective aperture and the TEM was switched back into image mode where a dark field image of the entire nanoparticle was recorded. The bright regions within the nanoparticle correspond to the nanoparticle areas that are aligned in such a way that they contribute to the selected diffraction reflex. This measurement shows that regions of substantially oriented substructures within the disc exist. EELS measurements show that incomplete discs, such as the diamond-shaped particle in Figure 3d, are of various non-stoichiometric phases with a compositional gradient. Non-aggregated areas, such as region 1, where crystallites are still visible, are of an iron-rich non-stoichiometric composition Co_1.1_Fe_1.9_O_4_. Regions with densely packed subunits, such as region 2, are of a cobalt-rich composition Co_1.8_Fe_1.2_O_4_.

**Figure 4:**
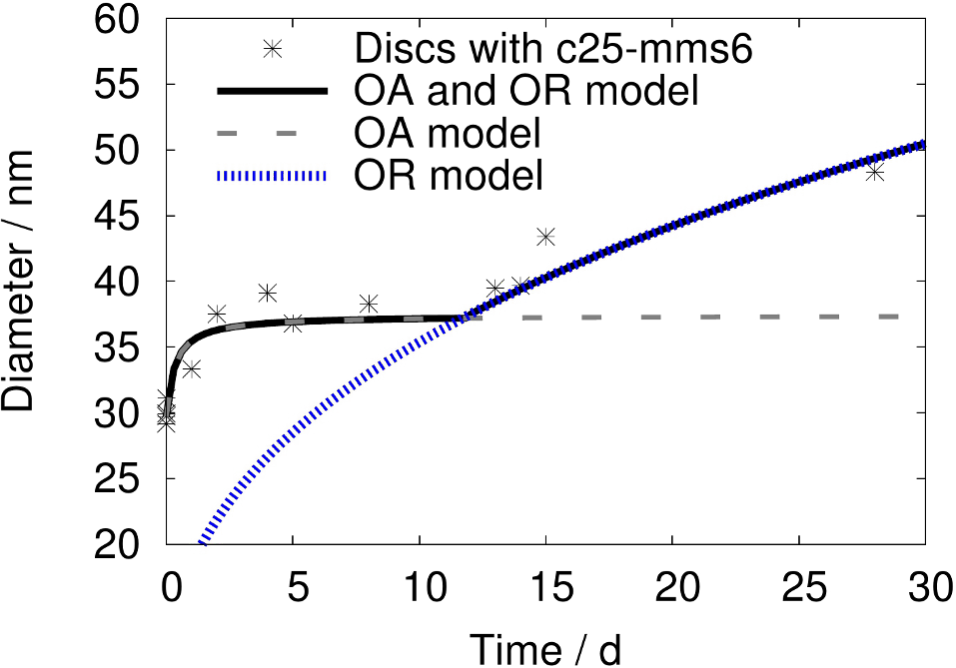
Change in the average disc diameter with time. The data is fitted with the kinetic model for an oriented attachment (OA) and Ostwald ripening (OR) process. The single contributions from the oriented attachment model as well as the Ostwald ripening are displayed as dashed lines.

Throughout the crystal growth process, several structural and compositional changes occur. A change towards a cobalt-rich phase with time, displayed in Table 1, indicates that several phase transformations take place during the growth process. Electron diffraction images of the discs between *t* = 5 min and *t* = 2 d, displayed at the top of Figure 3, show an increase in orientation with time. The electron diffraction patterns are direct representations of the reciprocal lattice of the nanoparticles. Sharp rings in the pattern as observed in the nanoparticle diffraction pattern after 5 minutes are caused by randomly oriented crystallites within the nanoparticle that lie in the same zone axis. The observed ring is diminished after 1 day showing that the crystallites start to orient along the same axis and that an increase in orientation with time occurs. Furthermore, a change in the zone axis and therefore top/bottom crystal face from [112] to [111] occurs after 2 days which indicates that a reorientation process takes place. In addition, the amount of irregular shapes is reduced by 2/3 in favor of hexagonal shapes during 28 days of particle growth while the amount of triangular shapes remains unaltered and is negligible (1%). The quantitative change in shape is displayed in Figure 2. The average diameter increases to *D*_av_ = 43 nm after a growth period of *t* = 15 d. The change in diameter with time is displayed in Figure 4. The smaller maximum, observed in the size distribution for the early stages of crystal growth, is diminished at the later stages of crystal growth (15 days), leading to a decreased variance (Figure 2) in average particle diameter. At the same time the larger maximum shifts to an increased diameter *D*_large_ = 40 nm. Tailing can now be observed in the size distribution as well, indicating that Ostwald ripening occurs in the later stages of particle growth. The diminishing amount of smaller nanoparticles and the increasing amount of larger particles leads to a non-symmetric size distribution (tailing) as observed after 15 days. The thickness of the discs was determined to be *z* = 10 nm by using the low loss spectrum that was obtained in the EELS measurements [23].

**Table 1:** Composition of diamond shaped nanoparticles at different stages of the growth process as determined by EELS.

Time	Phase

5 min	Co_1.1_Fe_1.9_O_4_
1 d	Co_1.3_Fe_1.7_O_4_
2 d	Co_1.6_Fe_1.4_O_4_
28 d	Co_2_FeO_4_

A final, stoichiometric Co_2_FeO_4_ particle, observed after 28 days of crystal growth, is displayed in Figure 3f. The HRTEM image of the tilted particle shows the monocrystallinity of the final, complete nanoparticle. The results suggest that nanoparticle growth in the bioinspired synthesis is a complex, multistep process, in which several structural and compositional changes occur.

## Discussion

Intermediate phases, such as oriented subunits within incomplete crystals as well as mesocrystal-like structures (Figure 3, bottom), were observed in addition to the final discs throughout the growth process. The intermediate phases are of non-stoichiometric compositions. A change towards a cobalt-rich phase can be observed with time, indicating that several phase transformations take place before Co_2_FeO_4_ discs are obtained, which supports our previous hypothesis [20]. Figure 4 shows that the change in diameter of the bioinspired synthesized particles agrees with the kinetic model for oriented attachment (OA) described by Zhang et al. [24] with Ostwald ripening (OR) dominating the later stage of nanoparticle growth. The change in diameter with time can be modelled by the proposed model [24]

[1]
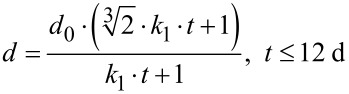


[2]
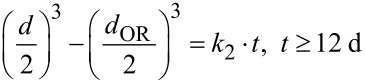


with *d*_0_ as the initial particle size and *k*_1_ = 3.4 × 10^−5^ s^−1^ as an oriented attachment kinetic reaction constant between two particles, *d*_OR_ as the particle diameter at which Ostwald ripening starts, *d* as the particle diameter with time, *k*_2_ = 6.11 Å^3^ s^−1^ as the rate constant and *t* as the time.

The vanishing smaller maximum in the size distribution (see Figure 2) is a further indication of an oriented attachment process. The smaller maximum at *D*_small_ = 5–20 nm corresponds to the size of the subunits, observed in the HRTEM measurements. These subunits coalesce during particle growth, leading to a decrease in variance as observed in Figure 2 (bottom). These substructures match the description of primary building blocks of an oriented attachment process. The majority of particles observed within the first minutes are irregularly shaped. The top/bottom crystal face of these particles was determined to (112). A change in shape towards a more distinct, hexagonal shape as well as the change in top/bottom crystal face from (112) to (111) occurs throughout the growth process. Those results, as well as the change in electron diffraction patterns with time indicate that an orientation process occurs during crystal growth.

**Figure 5:**
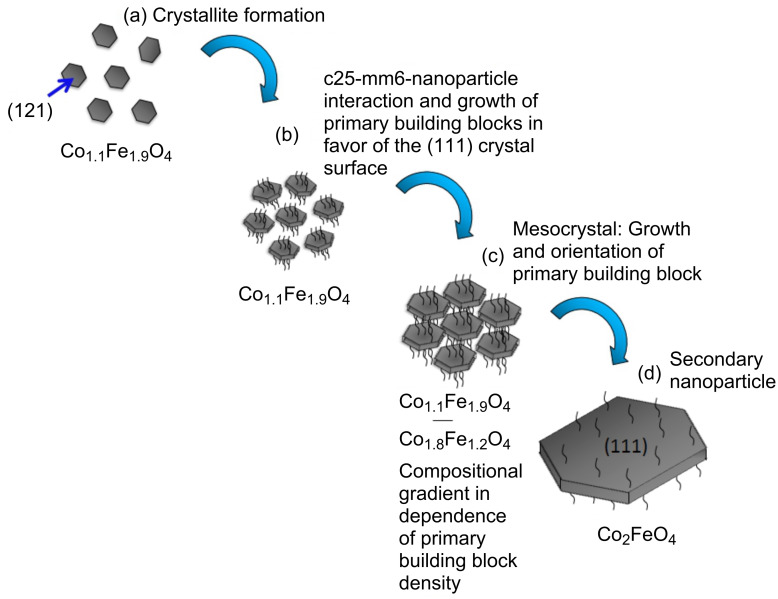
Schematic of nanoparticle formation during the biomimetic growth process. (a) Crystallites are formed, (b) c25-mms6 interacts with the crystallites which then grow into primary building blocks. (c) The primary building blocks assemble and reorient along a common crystallographic axis. (d) The primary building blocks aggregate to form the secondary nanoparticle.

Nanoparticle growth of the bioinspired cobalt ferrite particles matches the description of an oriented attachment process. Crystal growth via oriented attachment as well as mesocrystal formation have been described previously for biomineralization and biomimetic syntheses [19,25]. A schematic of this multistep process can be found in Figure 5. Nanoparticle growth via oriented attachment can be separated into the following four steps:

1. Crystallite formation
2. Polypeptide-nanoparticle interaction and growth of primary building blocks (pbb)
3. Mesocrystals: aggregation and orientation of primary building blocks
4. Formation of secondary nanoparticles

Only the growth of the cobalt ferrite nanoparticles was studied in this work. A detailed description of the crystallite formation is therefore not discussed here.

### Polypeptide-nanoparticle interaction and growth of primary building blocks

The driving force of crystal growth is the reduction of surface energy. The final crystal faces are therefore those with the lowest surface energy. Faces with higher energies have increased growth rates and vanish in the final morphology. The final top/bottom crystal faces of the cobalt ferrite particles, synthesized without c25-mms6, was determined to be (112) recently [20]. This crystal face was also found for the initially formed biosynthesized particles in this work. The change in top/bottom crystal face from (112) to (111), found in electron diffraction measurements, indicates that the energy of the (111) face is lowered. Prozorov et al. [22] came to a similar conclusion in their work. The change in surface energy can be explained if c25-mms6 interacts with the crystallites in such a way that growth in which the (111) final crystal face forms is favoured. Growth modification by peptide adsorption has previously been reported [9,21–22].

The polypeptide-stabilized crystallites assemble and form the intermediate phases. The assembly mechanism which leads to the formation of mesocrystals is discussed heavily in the scientific community at the moment. Several possible interaction mechanism have been proposed previously [9,11]. The interparticle forces, including van-der-Waals-attraction and repulsive forces due to c25-mms6 or hydration layers, are most likely the dominating factors in this process since the small substructures possess diameters below the superparamagnetic limit [9,11]. During the assembly, crystallites grow and form the subunits referred to as primary building blocks. They were observed in the HRTEM image of an irregularly-shaped disc (Figure 3).

### Orientation of primary building blocks

The primary building blocks align along a common crystallographic axis. Regions with specific orientations were observed in the dark field measurements in Figure 3. An increase in orientation with time was also observed in the change in electron diffraction images with time (Figure 3d). Such crystallographic reorientation within mesocrystals has been suggested previously [3,8–9,11].

### Formation of secondary nanoparticles

Mesocrystals have a large amount of energetically unfavourable inner surfaces and are therefore an unstable intermediate phase. To reduce the energetically unfavourable surfaces, the primary building blocks of which the discs are composed, join at specific crystallographic faces, leading to a reduced variance, as observed in Figure 2. To calculate the reduction of inner surfaces during the aggregation process, the number of primary building blocks (pbb) was calculated by

**Figure 6:**
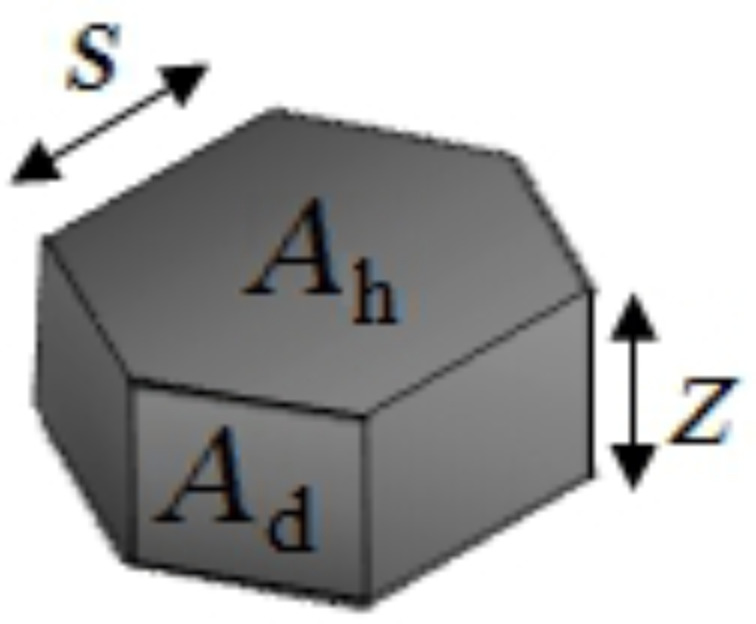
Geometry used for the calculation of the inner surface reduction, with *z* as the particle thickness and 2*s* = *D*_av_.

[3]
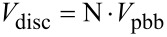


with 
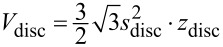
 as the disc volume, N as the number of primary building blocks and 
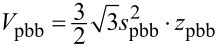
 as the volume of the primary building blocks. The volumes were calculated for the hexagonal geometry, displayed in Figure 6.

The number of primary building blocks within a single disc was calculated to be N = 417 for *z*_disc_ = 10 nm, *s*_disc_ = 22 nm, *z*_pbb_ = 1.7 nm, *s*_pbb_ = 2.5 nm. These values were found in the EELS and TEM measurements. To obtain the surface reduction by coalescence, the surface area of 417 primary building blocks was calculated and compared to the surface area of a secondary nanoparticle. The surface areas were calculated to *A*_disc_ = 6 · *A*_d,disc_ + 2 · *A*_h,disc_ and *A*_pbb_ = 6 · *A*_d,pbb_ + 2 · *A*_h,pbb_. The ratio

[4]
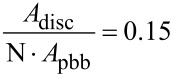


shows that the surface of the crystal is reduced by 85% during the aggregation process. The experiments show that during this aggregation process, a phase transformation towards the stoichiometric Co_2_FeO_4_ phase occurs. Such phase transformations have been discussed previously [12]. The lack of literature concerning the more cobalt-rich ferrite phase currently prevents a more detailed evaluation. A tertiary phase diagram of the system CoFeO at room temperature needs to be determined before a useful evaluation of the phase transformations can be conducted. Further theoretical studies are therefore required to understand the underlying principles of these transformations.

The fact that some mesocrystals remain after the aggregation of primary building blocks can be explained by the starting composition. Before the secondary particle is formed, a phase transformation from the iron-rich phase to cobalt-rich phase takes place. The initial starting composition is richer in iron though, while the particles are richer in cobalt. After 12 minutes, the cobalt precursor is substantially reduced, and iron-rich stoichiometric spheres form as a side product. Some particles cannot transform to the cobalt-rich phase and remain in the metastable mesocrystal state. Whether the final transformation to the stoichiometric cobalt-rich phase is enabled by a catalytic nature of the protein as previously proposed [20] or linked to the primary building block fusion requires further investigation. A possible polypeptide–nanoparticle interaction mechanism (micelle formation) has been proposed by Prozorov et al. [22]. However, this mechanism has not been verified yet and another interaction mechanism based on face-specific peptide adsorption is also possible and should be investigated. The fate of c25-mms6 during the fusion of the pbb cannot be deduced from the results. Whether the polypeptide is released from the pbb surface during the fusion process can only be answered once the interaction mechanism and interaction strength have been determined.

## Conclusion

The growth process of cobalt ferrite nanoparticles in a bioinspired synthesis, using the synthetic polypeptide c25-mms6, was studied. We were able to show that the growth of the particles, synthesized with c25-mms6, matches the description of an oriented attachment process. The change in nanoparticle diameter with time matches the kinetic model of an oriented attachment process, previously proposed by Zhang et al. [24]. The intermediate, metastable states, including primary building blocks and mesocrystals, were observed in this growth process. EELS and electron diffraction experiments show that a reorientation of the primary building blocks as well as several phase transformations occur prior to the formation of the secondary nanoparticles. However, the interaction between the polypeptide and the inorganic crystals is not yet understood and should be studied further to fully understand the biomineralization process.

## Experimental

### Synthesis of cobalt ferrite nanoparticles

The bioinspired synthesis utilizing the synthetic polypeptide c25-mms6 is described in detail in our previous work [20]. The particles obtained between 1 minute and 20 minutes as well as between 1 day and 28 days were studied. 2 μL particle suspension was dropcast onto a silicon-dioxide-coated copper TEM-grid from Plano GmbH. Excess solution was removed with filter paper. The sample was dried at room temperature afterwards.

### Microstructure

The microstructure and morphology were investigated using a Philips CM100 Transmission Electron Microscope (TEM) with an acceleration voltage *U* = 80 kV. The quantitative analysis of the nanoparticle sizes and shapes was performed manually by measuring the nanoparticle sizes in the TEM images using the program Scion. The amount of nanoparticles for each different shape was counted manually and normalized by the total amount of nanoparticles used for the statistical evaluation. A statistically sufficient amount of nanoparticles (>> 500) was used for the quantitative analysis. A FEI TECNAI F20 HRTEM with an acceleration voltage *U* = 200 kV was used for a detailed structural analysis. Electron diffraction measurements were also conducted with the FEI TECNAI F20. Since the distances and angles between the reflexes are material and zone axis specific, indexing the diffraction patterns yields information on the orientation and crystal faces of the different particles.

### Composition

The composition of the particles was studied by using EELS measurements, which are shown in detail in Supporting Information File 1. The FEI TECNAI F20 together with a Gatan GIF Tridiem spectrometer were used for these measurements to allow single particle composition measurements. In addition, the local composition gradient was measured by using a spectrometer entrance aperture to select regions with a diameter of ≈25 nm within a single nanoparticle. For the quantitative analysis, the pre-edge background was subtracted from each recorded spectrum. In order to subtract the pre-edge background, a power-law fit was performed (indicated by the dashed curves in the figures). Afterwards, the compositions of the samples were determined by using the Hartree–Slater method [26] and the signals shown as gray areas in the figures.

## Supporting Information

File 1 EELS spectra
