# Supplementary material for: Oriented attachment explains cobalt ferrite nanoparticle growth in bioinspired syntheses
Source: Beilstein J Nanotechnol. 2014 Feb 28;5:210–8. doi: 10.3762/bjnano.5.23 (PMC3943800; doi:10.3762/bjnano.5.23)
Supplement: File 1 — EELS spectra [file Beilstein_J_Nanotechnol-05-210-s001.pdf]

Supporting Information

for

Oriented attachment explains cobalt ferrite nanoparticle growth in bioinspired syntheses

Annalena Wolff*[1], Walid Hetaba[1,2], Marco Wißbrock[3], Stefan Löffler[2], Nadine Mill[1], Katrin Eckstädt[1], Axel Dreyer[4], Inga Ennen[1], Norbert Sewald[3], Peter Schattschneider[2,5] and Andreas Hütten[1]

Address: [1]Department of Physics, Bielefeld University, 33615 Bielefeld, Germany, [2](USTEM) University Service Centre for Transmission Electron Microscopy, Vienna University of Technology, 1040 Vienna, Austria, [3]Department of Chemistry, Bielefeld University, 33615 Bielefeld, Germany [4]Institute Advanced Cemarics, Hamburg University of Technology, 21073 Hamburg, Germany and [5]Institute of Solid State Physics, Vienna University of Technology, 1040 Vienna, Austria

Email: Annalena Wolff - awolff@physik.uni-bielefeld.de

* Corresponding author

EELS spectra

The EELS spectra which were used to determine the composition of the nanoparticles are displayed in Figures S1–S3. The solid lines indicate the spectrum as recorded. The signals which were used for the quantitative analysis are shown as gray areas in the Figures. For convenience, the EELS edges are labeled with the corresponding elemental symbols. The quantified EELS measurement of the cobalt ferrite particles after 5 minutes is given in Figure S1 while the quantified EELS measurement of the cobalt ferrite particles after 1 day is given in Figure S2. Figure S3 shows the quantified EELS measurement of the cobalt ferrite particles after 2 days.

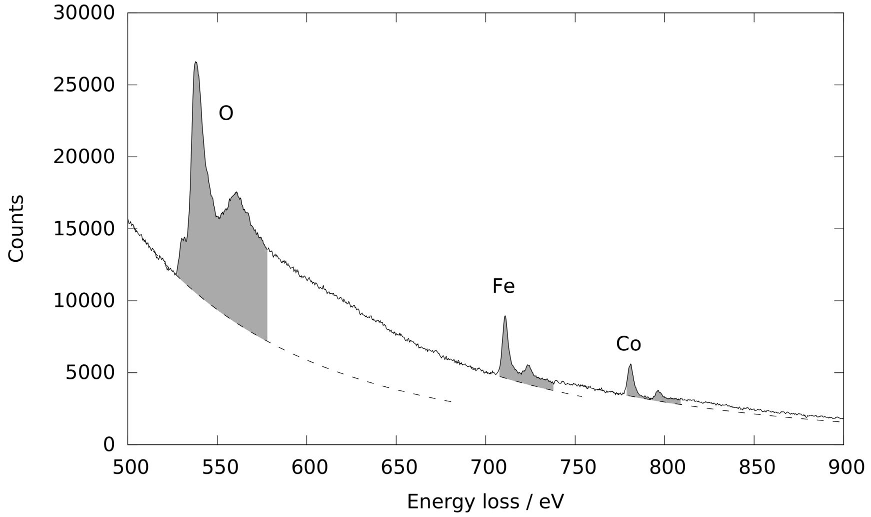

Figure S1: EELS measurement of the cobalt ferrite particles after 5 minutes. An exposure time of 20 seconds was used for the measurement.

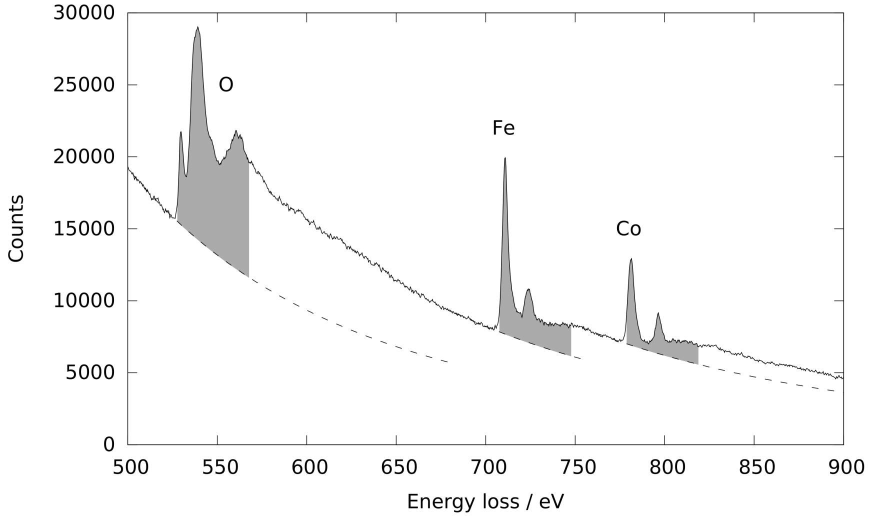

Figure S2: EELS measurement of the cobalt ferrite particles after 1 day. An exposure time of 10 seconds was used for the measurement.

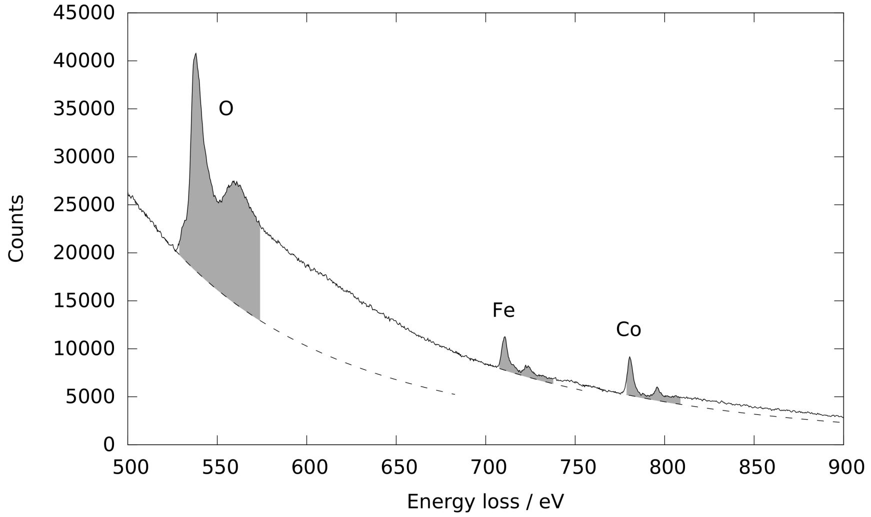

Figure S3: EELS measurement of the cobalt ferrite particles after 2 days. An exposure time of 15 seconds was used for the measurement.
